# Supplementary material for: Sex differences in febrile children with respiratory symptoms attending European emergency departments: An observational multicenter study
Source: PLoS One. 2022 Aug 3;17(8):e0271934. doi: 10.1371/journal.pone.0271934 (PMC9348645; doi:10.1371/journal.pone.0271934)
Supplement: S1 Fig — *Patients could have a identified viral co-infection. (PDF) [file pone.0271934.s001.pdf]

Phenotyping algorithm cause of infection

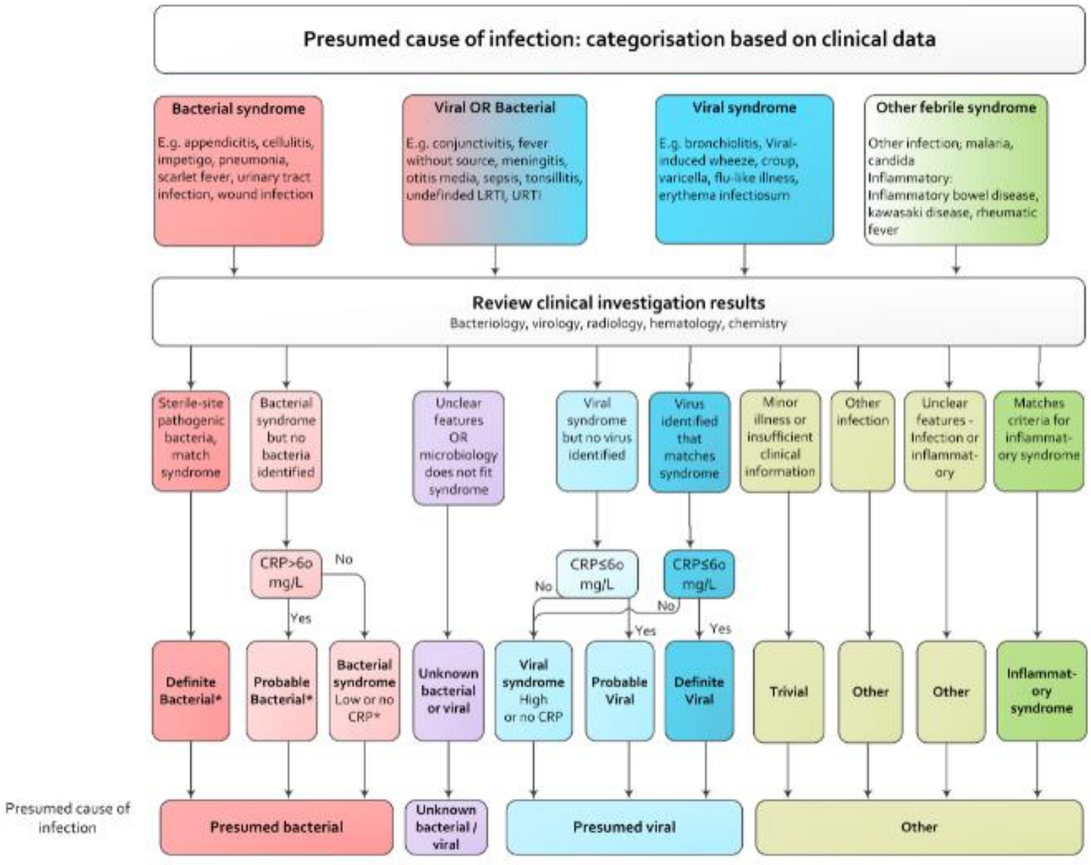

*Patients could have an identified viral co-infection.
