# Supplementary material for: Sex differences in febrile children with respiratory symptoms attending European emergency departments: An observational multicenter study
Source: PLoS One. 2022 Aug 3;17(8):e0271934. doi: 10.1371/journal.pone.0271934 (PMC9348645; doi:10.1371/journal.pone.0271934)
Supplement: S1 Table — Boys as reference group. Absolute numbers and percentages (%) are shown. (PDF) [file pone.0271934.s003.pdf]

Management stratified by sex with range per ED (N=19,781)

	**Boys (N=10,870 54%)**	**Range over EDs (%)**	**Girls (N=8911 46%)**	**Range over EDs (%)**
**Diagnostics**				
CRP	4204 (39)	7-91	3529 (40)	5-93
WBC	4193 (39)	8-91	3526 (40)	6-92
PCT	178 (2)	0-15	100 (1)	0-9
Respiratory test/culture	2113 (19)	2-34	1802 (20)	2-36
Blood culture	681 (6)	1-24	577 (7)	1-21
Chest X-ray	1850 (17)	5-31	1524 (17)	5-29
**Antibiotic treatment**	3316 (31)	20-38	2923 (33)	21-43
**Inhalation medication**	1399 (13)	5-21	883 (10)	5-18
**Oxygen therapy**	282 (3)	1-7	203 (2)	1-11
**Admission**	2214 (20)	4-53	1751 (20)	3-49

Boys as reference group.

Absolute numbers and percentages(%) are shown.
