# Supplementary material for: Sex differences in febrile children with respiratory symptoms attending European emergency departments: An observational multicenter study
Source: PLoS One. 2022 Aug 3;17(8):e0271934. doi: 10.1371/journal.pone.0271934 (PMC9348645; doi:10.1371/journal.pone.0271934)
Supplement: S2 Table — Boys as reference group. Adjusted for age, triage urgency, ill appearance, tachypnea, tachycardia, hypoxia, work of breathing, duration of fever, ED. (PDF) [file pone.0271934.s004.pdf]

Association between sex and management (N=19,781)

	Odds Ratio (95% CI)	Adjusted Odds ratio (95% CI)
**CRP/PCT/WBC**	1.04 (0.98-1.10)	0.97 (0.90-1.05)
**Respiratory test/culture**	1.05 (0.98-1.13)	1.02 (0.95-1.10)
**Blood culture**	1.04 (0.92-1.16)	1.05 (0.93-1.18)
**Chest X-ray**	1.01 (0.93-1.08)	1.03 (0.95-1.11)
**Antibiotic treatment**	1.11 (1.05-1.18)	1.08 (1.02-1.15)
**Inhalation medication**	0.75 (0.68-0.81)	0.82 (0.74-0.90)
**Oxygen therapy**	0.88 (0.73-1.05)	1.10 (0.88-1.38)
**Admission**	0.96 (0.89-1.03)	1.00 (0.92-1.08)

Boys as reference group.
Adjusted for age, triage urgency, ill appearance, tachypnea, tachycardia, hypoxia, work of breathing, duration of fever, ED.
