# Supplementary material for: Sex differences in febrile children with respiratory symptoms attending European emergency departments: An observational multicenter study
Source: PLoS One. 2022 Aug 3;17(8):e0271934. doi: 10.1371/journal.pone.0271934 (PMC9348645; doi:10.1371/journal.pone.0271934)
Supplement: S3 Table — Boys as reference group. Adjusted for age, triage urgency, ill appearance, tachypnea, tachycardia, hypoxia, work of breathing, duration of fever, ED. (PDF) [file pone.0271934.s005.pdf]

Association between sex and management in children with an upper respiratory tract infection (N=12,554)

	**Odds Ratio (95% CI)**	**Adjusted Odds ratio (95% CI)**
**CRP/PCT/WBC**	1.00 (0.93-1.08)	0.94 (0.85-1.04)
**Respiratory test/culture**	1.12 (1.02-1.22)	1.05 (0.96-1.16)
**Blood culture**	0.94 (0.77-1.15)	0.96 (0.78-1.18)
**Chest X-ray**	1.10 (0.96-1.25)	1.08 (0.94-1.24)
**Antibiotic treatment**	1.03 (0.95-1.11)	1.00 (0.92-1.08)
**Inhalation medication**	0.83 (0.71-0.97)	0.88 (0.75-1.05)
**Oxygen therapy**	0.89 (0.52-1.55)	1.12 (0.61-2.06)
**Admission**	0.96 (0.86-1.07)	1.00 (0.88-1.13)

Boys as reference group.

Adjusted for age, triage urgency, ill appearance, tachypnea, tachycardia, hypoxia, work of breathing, duration of fever, ED.
