# Supplementary material for: Sex differences in febrile children with respiratory symptoms attending European emergency departments: An observational multicenter study
Source: PLoS One. 2022 Aug 3;17(8):e0271934. doi: 10.1371/journal.pone.0271934 (PMC9348645; doi:10.1371/journal.pone.0271934)
Supplement: S4 Table — Boys as reference group. Adjusted for age, triage urgency, ill appearance, tachypnea, tachycardia, hypoxia, work of breathing, duration of fever, ED. (PDF) [file pone.0271934.s006.pdf]

Association between sex and management in children with a lower respiratory tract infection (N=3808)

	**Odds Ratio (95% CI)**	**Adjusted Odds ratio (95% CI)**
**CRP/PCT/WBC**	1.08 (0.95-1.23)	1.07 (0.89-1.29)
**Respiratory test/culture**	1.01 (0.86-1.20)	1.07 (0.89-1.29)
**Blood culture**	1.11 (0.90-1.37)	1.07 (0.84-1.36)
**Chest X-ray**	1.12 (0.99-1.28)	1.10 (0.95-1.27)
**Antibiotic treatment**	1.22 (1.07-1.38)	1.13 (0.98-1.30)
**Inhalation medication**	0.74 (0.65-0.84)	0.77 (0.66-0.89)
**Oxygen therapy**	0.97 (0.79-1.20)	1.09 (0.85-1.41)
**Admission**	0.90 (0.79-1.03)	0.91 (0.78-1.07)

Boys as reference group.

Adjusted for age, triage urgency, ill appearance, tachypnea, tachycardia, hypoxia, work of breathing, duration of fever, ED.
