# Supplementary material for: Sex differences in febrile children with respiratory symptoms attending European emergency departments: An observational multicenter study
Source: PLoS One. 2022 Aug 3;17(8):e0271934. doi: 10.1371/journal.pone.0271934 (PMC9348645; doi:10.1371/journal.pone.0271934)
Supplement: S5 Table — Boys as reference group. Adjusted for age, triage urgency, ill appearance, tachypnea, tachycardia, hypoxia, work of breathing, duration of fever, ED. (PDF) [file pone.0271934.s007.pdf]

Subgroup analysis in children below five years (N=14,967)

	**Boys (N=8334, 56%)**	**Girls (N=6633, 44%)**	**OR (95% CI)**	**Adjusted OR (95% CI)**
**CRP/PCT/WBC**	3287 (39)	2654 (40)	1.02 (0.96-1.09)	0.99 (0.90-1.09)
**Respiratory test/culture**	1404 (17)	1126 (17)	1.01 (0.93-1.10)	1.02 (0.93-1.11)
**Blood culture**	530 (6)	449 (7)	1.07 (0.94-1.22)	1.08 (0.94-1.24)
**Chest X-ray**	1417 (17)	1131 (17)	1.00 (0.92-1.09)	1.04 (0.95-1.15)
**Antibiotic treatment**	2419 (29)	2019 (30)	1.07 (1.00-1.15)	1.06 (0.98-1.14)
**Inhalation medication**	1207 (15)	739 (11)	0.74 (0.67-0.82)	0.80 (0.72-0.90)
**Oxygen therapy**	261 (3)	183 (3)	0.88 (0.72-1.06)	1.11 (0.87-1.40)
**Admission**	1897 (23)	1429 (22)	0.93 (0.86-1.01)	0.98 (0.90-1.08)

Boys as reference group.
Adjusted for age, triage urgency, ill appearance, tachypnea, tachycardia, hypoxia, work of breathing, duration of fever, ED.
