# Supplementary material for: Sex differences in febrile children with respiratory symptoms attending European emergency departments: An observational multicenter study
Source: PLoS One. 2022 Aug 3;17(8):e0271934. doi: 10.1371/journal.pone.0271934 (PMC9348645; doi:10.1371/journal.pone.0271934)
Supplement: S1 Checklist — (PDF) [file pone.0271934.s009.pdf]

STROBE Statement—checklist of items that should be included in reports of observational studies

	Item No.	Recommendation	Relevant text from manuscript
**Title and abstract**	1	(*a*) Indicate the study's design with a commonly used term in the title or the abstract	The study design is stated in the title and abstract.
		(*b*) Provide in the abstract an informative and balanced summary of what was done and what was found	The abstract is written according to *PLOS ONE* guidelines with all required information.
Background/rationale	2	Explain the scientific background and rationale for the investigation being reported	The background and rationale are described in the first three paragraphs of the introduction.
Objectives	3	State specific objectives, including any prespecified hypotheses	The objective is stated in the last paragraph of the introduction.
Study design	4	Present key elements of study design early in the paper	Key elements of the study design are presented in the first paragraph of the methods, and in detail explained in the consequent paragraphs.
Setting	5	Describe the setting, locations, and relevant dates, including periods of recruitment, exposure, follow-up, and data collection	The setting, location and relevant dates are described in the paragraphs 'Study population and setting' and 'Data collection' in the methods section.

Participants	6	(*a*) *Cohort study*—Give the eligibility criteria, and the sources and methods of selection of participants. Describe methods of follow-up<br>*Case-control study*—Give the eligibility criteria, and the sources and methods of case ascertainment and control selection. Give the rationale for the choice of cases and controls<br>*Cross-sectional study*—Give the eligibility criteria, and the sources and methods of selection of participants	Described in the paragraphs 'Study population and setting', in the methods section. There was no follow-up.
		(*b*) *Cohort study*—For matched studies, give matching criteria and number of exposed and unexposed<br>*Case-control study*—For matched studies, give matching criteria and the number of controls per case	Not applicable.
Variables	7	Clearly define all outcomes, exposures, predictors, potential confounders, and effect modifiers. Give diagnostic criteria, if applicable	Described in the paragraphs 'Data collection', 'Outcome measures', and 'Data analysis'.
Data sources/ measurement	8*	For each variable of interest, give sources of data and details of methods of assessment (measurement). Describe comparability of assessment methods if there is more than one group	Described in the paragraphs 'Data collection' and 'Data analysis'.
Bias	9	Describe any efforts to address potential sources of bias	Information bias was limited by entry guidelines for the eCRF, standardized data collection, training modules, feedback and teleconferences. Described in previous publication.
Study size	10	Explain how the study size was arrived at	This is a secondary analysis, described in previous publication.

Continued on next page

Quantitative variables	11	Explain how quantitative variables were handled in the analyses. If applicable, describe which groupings were chosen and why	Described in 'Data analysis'.
Statistical methods	12	(*a*) Describe all statistical methods, including those used to control for confounding	Described in 'Data analysis'.
		(*b*) Describe any methods used to examine subgroups and interactions	Described in 'Data analysis'.
		(*c*) Explain how missing data were addressed	Described in 'Data analysis'.
		(*d*) *Cohort study*—If applicable, explain how loss to follow-up was addressed<br>*Case-control study*—If applicable, explain how matching of cases and controls was addressed<br>*Cross-sectional study*—If applicable, describe analytical methods taking account of sampling strategy	Not applicable.
		(*e*) Describe any sensitivity analyses	Not applicable.
Participants	13*	(a) Report numbers of individuals at each stage of study—eg numbers potentially eligible, examined for eligibility, confirmed eligible, included in the study, completing follow-up, and analysed	The number of included participants is described in the first paragraph of the results section. All tables and figures included the number of patients that were analyzed.
		(b) Give reasons for non-participation at each stage	Not applicable.
		(c) Consider use of a flow diagram	-
Descriptive data	14*	(a) Give characteristics of study participants (eg demographic, clinical, social) and information on exposures and potential confounders	Characteristics of the study population are described in table 1, and in the first paragraph of the results section.
		(b) Indicate number of participants with missing data for each variable of interest	Missing data for each variable are described below table 1.
		(c) *Cohort study*—Summarise follow-up time (eg, average and total amount)	Not applicable.
Outcome data	15*	*Cohort study*—Report numbers of outcome events or summary measures over time	The number of patients with the outcome are reported in figure 1 until figure 3.
		Case-control study—Report numbers in each exposure category, or summary measures of exposure	
		Cross-sectional study—Report numbers of outcome events or summary measures	

Main results	16	(*a*) Give unadjusted estimates and, if applicable, confounder-adjusted estimates and their precision (eg, 95% confidence interval). Make clear which confounders were adjusted for and why they were included	Unadjusted odds ratios are provided in the supporting information files S2 Table, S3 Table, S4 table, S5 table. Adjusted odds ratios with 95% confidence intervals adjusted for age, disease severity, and ED are provided in the results section and figures 1 to 3.
		(*b*) Report category boundaries when continuous variables were categorized	Not applicable.
		(*c*) If relevant, consider translating estimates of relative risk into absolute risk for a meaningful time period	Not applicable.

Continued on next page

Other analyses	17	Report other analyses done—eg analyses of subgroups and interactions, and sensitivity analyses	Additional subgroup analyses were described in 'Data analysis' and results were shown in figure 2, figure 3 and S5 table.
**Discussion**			
Key results	18	Summarise key results with reference to study objectives	Key results are stated in the first paragraph and discussed in the subsequent paragraphs of the discussion section.
Limitations	19	Discuss limitations of the study, taking into account sources of potential bias or imprecision. Discuss both direction and magnitude of any potential bias	Limitations of the study are addressed in the paragraph 'Strengths and limitations' of the discussion section.
Interpretation	20	Give a cautious overall interpretation of results considering objectives, limitations, multiplicity of analyses, results from similar studies, and other relevant evidence	Interpretation of the results is described in the discussion.
Generalisability	21	Discuss the generalisability (external validity) of the study results	Generalizability is addressed in 'strengths and limitations' of the discussion section.
**Other information**			
Funding	22	Give the source of funding and the role of the funders for the present study and, if applicable, for the original study on which the present article is based	Title page.

*Give information separately for cases and controls in case-control studies and, if applicable, for exposed and unexposed groups in cohort and cross-sectional studies.

Note: An Explanation and Elaboration article discusses each checklist item and gives methodological background and published examples of transparent reporting. The STROBE checklist is best used in conjunction with this article (freely available on the Web sites of PLoS Medicine at http://www.plosmedicine.org/, Annals of Internal Medicine at http://www.annals.org/, and Epidemiology at http://www.epidem.com/). Information on the STROBE Initiative is available at www.strobe-statement.org.
