# Supplementary material for: Sex differences in febrile children with respiratory symptoms attending European emergency departments: An observational multicenter study
Source: PLoS One. 2022 Aug 3;17(8):e0271934. doi: 10.1371/journal.pone.0271934 (PMC9348645; doi:10.1371/journal.pone.0271934)
Supplement: Attachment — Submitted filename: Rebuttal letter_SexDifferences_PLOSONE.docx [file pone.0271934.s010.docx]

Manuscript reference number: PONE-D-22-05605
Title: Sex differences in febrile children with respiratory symptoms attending European Emergency Departments: an observational multicenter study

Dear dr. Emily Chenette and reviewers,

Thank you for reviewing our paper titled: "Sex differences in febrile children with respiratory symptoms attending European Emergency Departments: an observational multicenter study". We are very pleased to hear that a revised version of our manuscript is considered for publication in *PLOS ONE.* We sincerely thank the reviewers for their extensive comments to improve the paper, and have revised our manuscript according to their recommendations. The comments and suggestions on how to clarify the methodology and implications of our study were especially valuable. We hope that the revisions made to these sections made the overall paper more clear, and made it reproducible and more understandable for first-time readers. We feel that we were able to address all comments in the revised version and that our paper has improved significantly. Our point to point response to the individual review comments can be found below.

All co-authors have read and agreed upon the current submitted version.

Yours Sincerely, on behalf of all co-authors,

Prof. dr. H.A. Moll	dr. J.M. Zachariasse

**Additional journal requirements:**

**Comment**: Please ensure that your manuscript meets PLOS ONE's style requirements, including those for file naming.

**Answer:** Thank you. We have written our manuscript according to the PLOS ONE’s style requirements, including those for file naming.

**Comment:** You indicated that you had ethical approval for your study. In your Methods section, please ensure you have also stated whether you obtained consent from parents or guardians of the minors included in the study or whether the research ethics committee or IRB specifically waived the need for their consent.

**Answer:** We have added the following to the methods section: *“The need for informed consent was waived by the ethics committee.”*

**Comment:** We note that you have stated that you will provide repository information for your data at acceptance. Should your manuscript be accepted for publication, we will hold it until you provide the relevant accession numbers or DOIs necessary to access your data. If you wish to make changes to your Data Availability statement, please describe these changes in your cover letter and we will update your Data Availability statement to reflect the information you provide.

**Answer:** We will provide a DOI if the manuscript is accepted for publication.

**Comments from reviewer 1:**

In their paper, Sex differences in febrile children with respiratory symptoms attending European Emergency Departments: an observational multicenter study, the study authors aim to assess sex differences in presentation and management of febrile children with respiratory symptoms across twelve different European emergency departments in a roughly one-year period of time. The data set is quite large and finds statistically significant differences in both work of breathing on arrival and interventional therapies, but has some underlying issues with the methodology that need to be addressed.

**Major comments:**

**Comment 1.1:** Is there a reason that you didn’t just look at children with respiratory symptoms? Why combine fever and respiratory? I would put in a statement explaining this choice, given that there are many patients with URI/LRI who don’t also have fever.

**Answer 1.1:** We agree that we must clarify our choice. Our study was part of the MOFICHE study for which the inclusion criteria were children aged 0-18 years with fever (≥ 38 ℃) at the Emergency Department (ED) or with a history of fever (fever within 72 hours before ED visit). Therefore, our database only contain children with (history of) fever. The majority of these children had respiratory symptoms (63%) and a previous study regarding sex-specific differences in pediatric emergency care has shown that sex differences were present in children attending the ED with respiratory symptoms. Therefore, we decided to focus our study on children with respiratory symptoms in the population of febrile children. We have added the following to the study population paragraph in the methods section: “ *We decided to focus on febrile children with respiratory symptoms, since the majority of children had respiratory symptoms (63%) and a previous study regarding sex-specific differences in pediatric emergency care has shown that sex differences were present in children attending the ED with respiratory symptoms. (7)”*

**Comment 1.2:** The exclusion criteria should be re-explored. The authors excluded anyone with a co-morbidity, including asthma, but kept in patients who presented with other non-respiratory infections or neurologic complaints, such as seizure, which would seem to alter ER management and muddy the picture more than the presence of an underlying comorbidity. I think you need to decide to look at all-comers and then do subanalyses or decide you want to look to only at otherwise healthy children who are presenting with URI/LRI without another major presenting complaint. One of the main findings was that girls get more antibiotics overall, but this disappears when looking only at URI/LRI and can likely be explained by the higher prevalence of UTI – though we don’t have this data (only that girls have more co-infections of some kind). If you are truly looking at management of respiratory disease and trying to get at underlying sex differences, these kids need to be pulled out of the data.

**Answer 1.2:** Thank you for your interesting suggestions.

1. We agree that it would be interesting to analyse the children with comorbidity as well. However, children with comorbidity often receive more extensive management and therefore we cannot analyse management in the total population of children with and without comorbidity combined. Furthermore, comorbidity was defined as a chronic condition expected to last at least one year, which was categorized into tracti such as pulmonal, cardial, neurological. Unfortunately, we do not have detailed information on what comorbidities the children have and the categories are very broad. For example, pulmonal comorbidity could entail a large range of conditions such as asthma, bronchopulmonary dysplasia and cystic fibrosis. Thus it would not be feasible to perform subgroup analyses as detailed subgroups could not be defined. We have emphasized that we focused our study on previously healthy children and added the following to our conclusions in the abstract, in the results section and conclusion: “*Sex differences concerning presentation and management are present in previously healthy febrile children with respiratory symptoms presenting to the Emergency Department”. “We included 19,781 previously healthy febrile children with respiratory symptoms”. “In a large cohort of previously healthy febrile children with respiratory symptoms attending European EDs….”.* Additionally, our primary inclusion criteria were febrile children with respiratory symptoms. However, these children could have had other symptoms as well such as gastrointestinal and neurological symptoms. If we would include children with only respiratory symptoms we would lose a lot of cases and our results would be less representative of clinical practice, where for example young children with a respiratory tract infection often have both respiratory and gastrointestinal symptoms.

2. We agree that the higher antibiotic rate in girls could be explained by the higher prevalence of UTI in girls. Therefore, we have added UTI as a separate focus of infection group in table 1. In addition, we adjusted the following sentence in the methods section: *“The focus of infection was allocated retrospectively by the research team. We used the following categories: upper respiratory tract, lower respiratory tract, urinary tract and other (gastrointestinal tract, childhood exanthemas/flu-like illness, soft tissue or skin/musculoskeletal, sepsis/meningitis, undifferentiated fever and inflammatory illness).”* The prevalence of UTI in our study population was 1.4% (281/19,781). UTI was more frequently the focus of infection in girls than in boys 2.4% (217/8911) vs 0.6% (64/10,870), with antibiotic treatment in 91% (198/217) in girls and in 89% (57/64) in boys. We have performed a sensitivity analyses in our study population after excluding children with UTI as focus of infection, which showed no significant difference in antibiotic treatment in boys and girls with an aOR 1.03 (95% CI 0.97-1.10). However, it seems not realistic to exclude children with UTI since our inclusion criteria is based on presenting symptoms, namely respiratory symptoms. Methodological it seems not feasible to exclude children based on their focus of infection, since focus of infection is based on physical examination and diagnostics. We added the following to the data analysis section: *“Based on our results, we performed an additional sensitivity analysis with exclusion of children with the urinary tract as focus of infection.”* In the discussion section we added the following: *“This might be explained by a higher prevalence of a urinary tract infection in girls than boys (2.4% vs 0.6%). A sensitivity analysis where we excluded children with a urinary tract infection (N=281) did not show a significant difference in antibiotic prescription in girls and boys (aOR 1.03, 95% CI 0.97-1.10).”* In the results section of our abstract we added the following: *“Girls received less inhalation medication than boys (aOR 0.82, 95% CI 0.74-0.90), but received antibiotic treatment more frequently than boys (aOR 1.09, 95% CI 1.02-1.15), which is associated with a higher prevalence of urinary tract infections.”*

**Comment 1.3:** I would clarify further the purpose of this study – it’s hard for me to piece together the clinical significance. The last sentence of the introduction says that finding differences in how these children present “may improve diagnosis and treatment” – but there is no further discussion of this and it’s hard to say how this data would do this. The last sentence of the discussion seems more reasonable – that there are differences in care and next step is to determine if these are based on clinical symptoms or physicians’ attitudes (ie, some type of implicit bias). I think I would stick with this theme, that this is purely descriptive, but more work needs to be done to figure out why there are differences.

**Answer 1.3:** Thank you for your suggestion. We agree that the purpose of you study is not to improve diagnosis and treatment, but to examine whether there are sex differences and that the next step is to determine if these are based on clinical symptoms or physicians’ attitude. We have adapted the last sentence of our introduction as following: *“Insight in these sex differences may increase our understanding on the role of sex in pediatric emergency medicine and whether these are based on clinical symptoms or physician’s attitude.”*

**Minor comments:**

*Abstract*

**Comment 1.4:** Line 83: I’d include p values (or ORs) with these statistics, particularly since the differences are so small.

**Answer 1.4:** We agree that we should add p values since the differences are so small. We added the following to the results section of the abstract: *“Girls present less frequently with tachypnea (15% vs 16%, p=0.002) and increased work of breathing (8% vs 12%, p<0.001)”*

*Introduction*

**Comment 1.5:** Line 97: Small point, but I’d replace “inequality” with “inequity” – there are many reasons that different groups receive different treatments, but the goal should be to provide equitable care – which is what I think you are trying to get at here.

**Answer 1.5:** Thank you for your suggestion. We agree that we should replace inequality with inequity and have adjusted this sentence in the introduction: *“Identifying these differences is crucial for not only optimizing health care outcomes but also minimizing existing inequity in health care.”*

**Comment 1.6:** Line 100: Would change “adults in emergency medicine” to “adults presenting to the emergency department”

**Answer 1.6:** We adjusted the following sentence: *“Several studies have been conducted regarding sex differences in adults presenting to the Emergency Department (ED), finding clinically relevant differences.*

**Comment 1.7:** Line 107: “research on sex differences in children remains scarce” – I don’t think this is true. If you look at the reference list for the next paper you talk about, there are multiple studies there. Much of the research coming out of pediatric EM will contain demographic data and comment on any sex difference in presentation. You may be able to say that we need more research into differences in emergency management by sex – since I agree, there’s not as much out there about management in particular.

**Answer 1.7:** We agree that we particularly want to focus on sex differences in management of febrile children with respiratory symptoms. Therefore, we have specified this and adjusted the following sentence: *“…research on sex differences in management of children remains scarce, especially in emergency medicine.”*

**Comment 1.8:** Line 108: “The first study to assess the role of sex in pediatric emergency…” I would definitely talk more about this study – ie, what they found. I think it leads nice into your study and puts it into more context. They looked into a huge group of kids and found that boy present more frequently to the ED, get more inhalational medications, and girls get more labs – but this was all pooled data and didn’t look at a specific population. Here, you are attempting to only look at febrile kids with respiratory symptoms so you can really get to whether or not there are differences that exist here. This will also make your next sentences about sex different in asthma/bronchiolitis make more sense since they seem a bit disconnected currently.

**Answer 1.8:** Thank you for your suggestion. We adjusted the following sentence in the introduction: *“This study was conducted in a large heterogeneous population of children using pooled data and found that boys present more frequently to the ED and receive inhalation medication more often when presenting with respiratory symptoms.”*

**Comment 1.9:** Line 123: “may improve diagnosis and treatment” – this doesn’t seem to be the goal of your paper overall based on the discussion and conclusions. I think you mean the role of sex in the management of children in the emergency department. There is no discussion in the paper at all around improving diagnosis or improving treatments since there is no information on outcomes (ie did they receive a correct diagnosis or was that treatment useful), only descriptive information on who presented and what was done.

**Answer 1.9:** We totally agree and have adjusted this part of the introduction. See comment and answer 1.3.

*Methods*

**Comment 1.10:** Line 150: “We excluded children with missing data on management and children with comorbidity.” Two things

1. I would move your source for what defines a comorbidity here. It looks like the source is referencing a paper that uses ICD-9 codes (though your study was done during the ICD-10 era, so I would use an updated source – looks like there have been two newer versions, PMID: 29496546). Were these codes only present at the index visit? Or if the patient ever had this code?

2. Why did you exclude these children? Seems like if these are large university hospitals you are going to have a lot of kids with comorbidities. Particularly in a study that is looking at the use of inhalational medications which should be mostly used in children who carry a diagnosis of asthma (or should). Is it possible to include them and then do a subanalysis of children who did not have comorbidities? Or look at all the data and then control for the number of kids with asthma? If not – I would at least include information on how many kids (what %) you excluded due to a comorbidity.

**Answer 1.10:** Thank you for your suggestions.

1. We have moved the definition of comorbidity to the part where we mention the exclusion criteria as followed: *“We excluded children with missing data on management and children with comorbidity, which was defined as having a chronic condition expected to last at least one year.”* The research team at each participating ED extracted data on comorbidity as stated in the ED charts or previous history forms. We have added this to the following sentence: *“Data collected included age, sex, triage urgency, comorbidity (as stated in the ED charts or previous history forms)…”.* Additionally, we have updated the reference to PMID 29496546.

2. Of the febrile children with respiratory symptoms, 17% (4111/24,380) had one or more comorbidities, with a broad range of 5.5% to 65.4% per ED. The participating EDs are part of large university or teaching hospitals. Therefore our results are more generalizable to general hospitals when we exclude the children with comorbidity, since the prevalence of comorbidity in these hospitals would be lower or comparable with hospitals in our study with lowest prevalence of comorbidities. The pulmonal comorbidity group entails 26% (1056/4111) of all the children with comorbidity Unfortunately, we do not have information on which pulmonal comorbidity children had and cannot perform a subgroup analysis in children with asthma.

**Comment 1.11:** Line 160: “Children could have had gastrointestinal (diarrhea or vomiting) or neurological (seizures or focal neurological signs or meningeal signs) symptoms in addition to their respiratory symptoms.” Were they allowed to have other symptoms as well? Or were they excluded if they had other symptoms except fever, respiratory and possibly GI/neuro. I would clarify what you mean here.

**Answer 1.11:** We agree we should clarify this. It was allowed to have multiple symptoms on presentation. As part of the MOFICHE data collection we restricted data on presenting symptoms to prespecified complaints including respiratory (runny nose, sore throat, coughing, sneezing), gastrointestinal symptoms (diarrhea, vomiting), and neurological symptoms (seizures or focal neurological signs or meningeal signs). Children could have had other symptoms such as rash or urogenital complaints, but these data were not collected in MOFICHE. We have adjusted the following sentence in the data collection paragraph: “*Data on presenting symptoms were restricted to prespecified complaints including respiratory, gastrointestinal (diarrhea, vomiting) and neurological symptoms (seizures or focal neurological signs or meningeal signs), and children were allowed to have multiple presenting symptoms.”*

**Comment 1.12:** Line 174: “We categorized diagnostic tests into simple and advance.” This classification seems a bit arbitrary – there are many kids with fever/respiratory who get a CXR and go home on antibiotics. I wouldn’t consider that more advanced than doing bloodwork (which is often reserved for kids who are actually sicker since it’s a more invasive intervention). I would get rid of this distinction and just split into “imaging,” “bloodwork,” and “respiratory test/culture”.

**Answer 1.12:** We agree that our categorization of the diagnostic tests is a bit arbitrary and that the wording simple and advanced might be setting dependent. We have split the diagnostic tests in WBC/CRP/PCT, respiratory test/culture, blood culture and chest X-ray. We adjusted the following sentence: “*We categorized diagnostic tests into general bloodwork (CRP, white blood cell count (WBC) and procalcitonin (PCT)), respiratory test/culture, blood culture, and chest X-ray”.* This new categorization of diagnostics tests did not significantly influence any of the results. Additionally, we have updated all the tables and figures with this new categorization of diagnostic tests.

**Comment 1.13:** Line 186: How was ill appearance defined? Triage nursing? Physicians note?

**Answer 1.13:** The research teams received the following instructions: “Classify ill, moderately ill, irritable and uncomfortable as ill.” This data could have been extracted from triage nursing of physicians notes. We have added the following to the methods section: “*We adjusted for the following covariates: age, triage urgency, ill appearance (classify as ill if ill, irritable or uncomfortable is stated in the chart written by triage nurses or physicians)…”.*

**Comment 1.14:** Line 193: It’s unclear to me what “behavioral and psychological processes” means and has to do with fever/respiratory symptoms and ED management. Please clarify what you mean here.

**Answer 1.14:** We agree that ‘behavioral and psychological processes’ in the text is confusing. We should clarify this. Pubertal sex hormones can cause changes in behaviour of children, which could influence the decision making of the clinicians regarding management at the ED. However, behaviour can be influenced by other factors as well such as the child’s environment. Therefore, we decided to leave out the wording behaviour since it is a complex process with a lot of factors involved. We adjusted the following sentence*: “Subsequently, we performed a subgroup analysis in children up to five years to reduce the influence of pubertal sex hormones, since the rise of adrenal androgens occurs around the age of six to eight years.”*

**Comment 1.15:** Line 194: Please change odds ratios to adjusted odds ratios

**Answer 1.15:** We agree and have changed this. *“Adjusted odds ratios (aORs) were calculated and a 95% confidence interval (CI) was given.”*

*Results*

**Comment 1.16:** Line 205: Please include the p value for these numbers, particularly since they are so similar between groups. Or, even better, include the odds ratio with the confidence interval.

**Answer 1.16:** Thank you for your suggestion. We have added the p values of the significant differences in the text, since we agree that these are small differences. “*Girls had less frequently tachypnea (15% vs 16%, p=0.002) and increased work of breathing (8% vs 12%, p<0.001) compared with boys, whereas girls had more often tachycardia (28% vs 23%, p<0.001).”*

**Comment 1.17:** Table 1:

I think you should include the actual p values here instead of just the asterisk that it’s <0.05. Again – I think having an odds ratio with a confidence interval would also provide much more information than a p value. I also can’t tell what is significant – eg focus of infection is labeled as significant, but I can’t tell if one sex is more likely to have an “other” focus of infection, which will likely affect management. Same with cause of infection.

**Answer 1.17:** Thank you for your suggestion. Since the table is a descriptive overview of our study population and we have not corrected for confounders, we decided to not calculate odds ratio’s with confidence intervals. Additionally, we have not adjusted our main analysis for focus and cause of infection, which were determined by the research team after the ED visit. As mentioned in comment and answer 1.16, we have added the exact p values in the text. The additional p values of the variables with a significant different in table 1 are: triage urgency p=0.004, focus of infection p <0.001, cause of infection p<0.001. We performed chi-squared tests for categorical variables in table 1 and a significant difference only means that there are differences in focus of infection and cause of infection between boys and girls for some of the categories.

**Comment 1.18:** Table 2/Figure 1.

I would combine this with Figure 1. It looks strange to have this table without any statistics on the significance – you have to then go to Figure 1 to find the actual aORs – but Figure 1 doesn’t include all of the variables in Table 2… so I still don’t know what the aOR of getting a CXR was. It’s a pretty figure, but I think it makes more sense to just add this information to a fourth column in Table 2 if editor agrees.

I would control for presence of another source of infection other than respiratory. This is certainly going to change management. As you say – more females had UTIs and they are going to then get antibiotics that are not for their respiratory disease. Instead of just adding as a limitation, you can control for this, particularly since one of your main findings that barely reached significance was antibiotic use. You should also consider removing these patients (making it an exclusion criteria) since this will so strongly affect management (I think this is the better option).

As a side note – the number of children who are getting interventions seem really high to me. From your S1_Table – it looks like one of your EDs sends blood work on 93% of the patients it sees with fever/respiratory symptoms which seems outrageous to me, particularly since you include any rhinorrhea or sore throat as a respiratory symptom and most of these patients are low-acuity. Is this correct? It feels like it can’t possibly be the same patient population.

**Answer 1.18:** Thank you for your suggestions.

1. Table 2 is a descriptive overview of the management performed with more details than figure 1. We did not perform statistical tests in table 2 since it is not informative when it is not adjusted for confounders yet. The purpose of table 1 and table 2 is merely to show the readers how our data looks like. Therefore, we think it is better to not combine table 2 and figure 1.

2. We think that it is not feasible to correct for focus of infection or to exclude children with UTI, since in clinical practice you do not know whether a child has a UTI at presentation. Please also see comment and answer 1.2 where we did perform a sensitivity analysis after excluding children with UTI.

3. We agree that the range over EDs varied and we can confirm this is correct. It is a nice representation of diversity in management in EDs in Europe, which is probably also different from the US. We think management performed will depend on setting, type of hospital, whether there is a general practitioner available, and what is considered normal for the specific setting. A previous publication on the MOFICHE database has shown that large diversity is present between the participating settings (PMID: 31338429). Furthermore, we only collected data on prespecified symptoms such as rhinorrhea and sore throat but these children could have had more severe symptoms as well during physical examination such as increased work of breathing which is shown in table 1.

*Discussion*

**Comment 1.19:** Line 264: “This is in line with a previous study in which girls received less inhalation medication compared with boys in children with respiratory symptoms attending the ED (pooled OR 0.79, 95% CI 0.73 to 0.86).” Please move your reference for these statistics to after this line.

**Answer 1.19:** We have moved our reference.

**Comment 1.20:** Line 270: I’m having some trouble with this argument – please clarify here. It seems like you are saying that boys are more likely to wheeze, which is why they get more inhalational therapies – but then say this can’t be because we excluded kids with asthma. I think the truth is that if you are getting bronchodilators and are over the age of 2, you are going to get a diagnosis of asthma (since having wheeze/cough responsive to bronchodilators is essentially the definition). The kids in this study who received bronchodilators probably fell into one of two categories – either they just hadn’t had an ICD-10 code for asthma put on their chart yet, or they were under 2 years old and wheezing, so had a tentative diagnosis of viral induced wheeze and may or may not later be diagnosed with asthma. Either way – both of these are more prevalent in males (as you point out), which likely explains why they are getting more inhalational therapy.

**Answer 1.20:** We agree that boys have more often asthma and wheeze more frequently and therefore receive more inhalation therapy than girls. We will rephrase the sentence as following: *“However, children with comorbidity including asthma were excluded in our study, so this group does not contribute to the sex-specific differences.”*

**Comment 1.21:** Line 274: Agree with this argument for more antibiotics in girls – but this is why you should exclude these patients with other reasons to get antibiotics if you really want to looks at management of respiratory complaints.

**Answer 1.21:** Thank you for your comment. Please see comment and answer 1.2 and 1.18. Additionally, it is not possible to exclude children who received antibiotics for other causes than respiratory tract infection since we do not have information on the reason why antibiotics were prescribed.

*Other Figures*

**Comment 1.22:** See comments in results for Tables 1&2 and Figure 1

**Answer 1.22:** We have added headings to the results section to emphasize the descriptive part and the main analyses part concerning the association between sex and management. *“Description of the study population.” “Association between sex and management”.* Please also see comments and answers 1.17 and 1.18.

**Comment 1.23:** I don’t think you need supplemental tables 2-4 – they are the same as Figures 1-3 with the inclusion of the unadjusted odds ratio, which you don’t need to include – though will leave to editor.

**Answer 1.23:** We agree that the adjusted odds ratios are similar to figures 1-3. However, for now we kept the unadjusted odds ratios in the supplemental tables for readers who are wondering whether the odds ratios have changed after adjustment for confounders.

**Comment 1.24:** No comments on other figures/tables

**Comments from reviewer 2:**

The authors have performed an observational study evaluating the association of sex with characteristics of children visiting Emergency Departments for febrile respiratory illness. Strengths of the study include the inclusion of multiple centers, large number of subjects, and depth of available data.

**Comment 2.1:** Overall, the expected implications and intended applications of the study findings are unclear. Were the authors intending the inform the incorporation of patient sex into clinical decision making or even the development of a clinical decision rule? Or were they intending to shed light in potential sex-based disparities in emergency care, particularly with respect to management? There are parts of the introduction that suggest either objective. The authors' proposed significance of this study could be stated more clearly in the introduction and discussion.
**Answer 2.1:** Thank you for your comment. We agree that we should clarify the expected implications and intended applications of our descriptive study. The purpose of our study is indeed to shed light on potential sex-based differences in emergency care, particularly with respect to management. The next step would be to determine if these are based on clinical symptoms or physicians’ attitude. We have adapted the last paragraph of our introduction and added the following sentence: *“Insight in these sex differences may increase our understanding on the role of sex in pediatric emergency medicine and whether these are based on clinical symptoms or physician’s attitude.”* Additionally, in the discussion section we mentioned that this study is a first step in providing insight in the role of sex in management in febrile children with respiratory symptoms in emergency medicine. Future research should focus on to what extend the observed differences between boys and girls are related to clinicians’ attitudes or to differences in clinical symptoms at the time of presentation and disease severity. See also comment and answer 1.3.

*Abstract*
**Comment 2.2:** General: The conclusions are largely a reiteration of the results and instead could be nbetter used to discuss the implications of the results.
**Answer 2.2:** We agree that we should adapt the conclusion of the abstract to discuss the implications of the results. We have changed this as following: *“Sex differences concerning presentation and management are present in previously healthy febrile children with respiratory symptoms presenting to the Emergency Department. Future research should focus on whether these differences are related to clinicians’ attitudes, differences in clinical symptoms at the time of presentation and disease severity.”*

**Comment 2.3:** 70: "0-18 years" could be interpreted to be inclusive of 18-year-olds, whom the study presumably excluded based on the age groups in Table 1.
**Answer 2.3:** Thank you for your comment. We have adapted this through the whole manuscript as following: *“children aged 0 - <18 years”*

**Comment 2.4:** 79: Could mention that subgroups analyses were performed by upper versus lower respiratory tract infection
**Answer 2.4:** We have added she specific subgroup analyses as following: *“Additionally, subgroup analyses were performed in children with upper and lower respiratory tract infections and children below five years.”*

**Comment 2.5:** 83: See comment on 205.
**Answer 2.5:** We agree that the sex difference in tachypnea is small and therefore have added the exact p value in the results section of the abstract and manuscript as following: *“Girls present less frequently with tachypnea (15% vs 16%, p=0.002) and increased work of breathing (8% vs 12%, p<0.001) compared with boys.”* Since the difference is too small to be considered clinically relevant, we have adjusted our conclusion of the abstract and deleted the sentence regarding girls having less frequently tachypnea.

*Methods*
**Comment 2.6:** 144: See comment on 70.
**Answer 2.6:** See comment and answer 2.3.

**Comment 2.7:** 146: Why were shortness of breath, tachypnea, or wheezing not included as respiratory symptoms? These symptoms would capture children with greater illness severity who would be of higher interest clinically to pediatric emergency physicians than those with only fever and sore throat.
**Answer 2.7:** Thank you for your comment. As part of the MOFICHE data collection we restricted data on presenting symptoms to prespecified complaints including respiratory (runny nose, sore throat, coughing, sneezing), gastrointestinal symptoms (diarrhea, vomiting), and neurological symptoms (seizures or focal neurological signs or meningeal signs). We have adjusted the following sentence in the data collection paragraph: “*Data on presenting symptoms were restricted to prespecified complaints including respiratory, gastrointestinal (diarrhea, vomiting) and neurological symptoms (seizures or focal neurological signs or meningeal signs), and children were allowed to have multiple presenting symptoms.”* Unfortunately, we did not have data on wheezing as we mentioned as limitation in the discussion section. Furthermore, the majority of children with increased work of breathing and tachypnea presented with respiratory symptoms. We analysed at your suggestion how many children would be included if we added increased work of breathing and tachypnea as inclusion criteria, based on the items collected from the physical examination, and this was less than 1% of our study population. Therefore, we included children based on their presenting symptoms at ED presentation and not based on a very selected group with findings on physical examination.

**Comment 2.8:** 151: Exclusion of children with comorbidity needs to be explained more. What level of comorbidity was excluded, complex illness or all chronic conditions including even mild intermittent asthma? If all children who have received a diagnosis of asthma are excluded, it should be noted that there is significant variability as to which children with prior respiratory illnesses have received a chronic diagnosis of asthma versus only acute diagnoses of "bronchiolitis," "wheeze," etc. that may confound the study results.
Given the high prevalence of asthma and other chronic conditions in among children, the exclusion of children with comorbidity significantly limits the applicability of these results to the overall ED population. If this is intentional, the authors could be more explicit in stating that their study focuses only on previously healthy children.
**Answer 2.8:** Thank you for your comment. We agree that children with comorbidity are an important subgroup of children in EDs of teaching and university hospitals. However, children with comorbidity often receive more extensive management and therefore we cannot analyse management in the total group of children with and without comorbidity combined. In our study, comorbidity was defined as as a chronic condition expected to last at least one year, which was categorized into tracti such as pulmonal, cardial, neurological. Unfortunately, we do not have detailed information on what comorbidities the children have and the categories are very broad. For example pulmonal comorbidity could entail a large range of conditions such as asthma, bronchopulmonary dysplasia and cystic fibrosis. Therefore, it was not feasible to perform subgroup analyses since we had no detailed information on what kind of pulmonal comorbidity the child had. In the febrile children with respiratory symptoms, we excluded 17% (4111/24,380) children with one or more comorbidities with a broad range of 5.5% to 65.4% per ED. Therefore our results are more generalizable to general hospitals when we exclude the children with comorbidity, since the prevalence of comorbidity in these hospitals would be lower or comparable with hospitals in our study with lowest prevalence of comorbidities. The pulmonal comorbidity group entails 26% (1056/4111) of all children with comorbidity. We agree that we should emphasize that we focused on previously healthy children. We have added this to the conclusions of the abstract, in the results section and conclusion: *“Sex differences concerning presentation and management are present in previously healthy febrile children with respiratory symptoms presenting to the Emergency Department”.* *“We included 19,781 previously healthy febrile children with respiratory symptoms”. “In a large cohort of previously healthy febrile children with respiratory symptoms attending European EDs….”.* See also comments and answers 1.2 and 1.10.

**Comment 2.9:** 164: How were children with concurrent infections allocated? Given that the percentages in Table 1 add up to 100%, presumably only one focus of infection was assigned to each child, but if one were to present with influenza and urinary tract infection, both are clinically significant and worth accounting for. The categories perhaps should not be treated as mutually exclusive.
**Answer 2.9:** Thank you for your question. We confirm that children could only have one focus of infection ticked in our database. However, when the box ‘focus of infection other’ was ticked by the research team, details could be given. For example the research team could mention that a child had both a gastrointestinal infection and respiratory infection, but this was only the case in a small number of children. In the majority of the cases the most clinically relevant focus of infection was ticked. We added below table 1:” *^¥^ Most clinically relevant focus of infection was assigned.”* Additionally, the PERFORM flowchart was created that bacterial infections had priority over viral infections in case of a mixed infection and therefore the percentages in table 1 add up to 100% as well for cause of infection.

**Comment 2.10:** 187: Unclear what is meant by ED setting.
**Answer 2.10:** We agree that we should rephrase this. We meant participating EDs and therefore have adjusted this sentence in the methods as following: *“We adjusted for the following covariates: age, triage urgency, ill appearance (classify as ill if ill, irritable or uncomfortable is stated in the chart written by triage nurses or physicians), duration of fever, vital signs (tachycardia, tachypnea, hypoxia), increased work of breathing and participating ED”.*

*Results*
**Comment 2.11:** 200: In addition to the study sample size, this sentence should include the total initial number of febrile children with respiratory symptoms and the number that were excluded due to missing data or presence of morbidity.
**Answer 2.11:** Thank you for your suggestion. We have added the following to the results section: *“We included 19,781 previously healthy febrile children with respiratory symptoms after excluding 18% (4321/24,380) of the children due to comorbidity and subsequently excluding 1% (278/20,059) due to missing data on outcome measures.”*

**Comment 2.12:** 205: The statement that girls had lower frequency of tachypnea is repeated multiple times across the manuscript but is questionable. The difference by sex, while perhaps statistically significant with the large sample size, is too small to be considered clinically important.
**Answer 2.12:** We agree that the sex difference in tachypnea is small and therefore have added the exact p value in the results section of the abstract and manuscript as following: *“Girls present less frequently with tachypnea (15% vs 16%, p=0.002) and increased work of breathing (8% vs 12%, p<0.001) compared with boys.”* Since the difference is too small to be considered clinically relevant, we have adjusted our conclusion of the abstract and deleted the sentence regarding girls having less frequently tachypnea.

*Discussion*
**Comment 2.13:** General: The entirety of the discussion is in one large paragraph and may be more easily readable in divided into multiple paragraphs by topic.
**Answer 2.13:** Thank you for your suggestion. Based on your comment 2.14, we have shortened the paragraph of our main findings in the beginning of the discussion section. Because the discussion now consists of 3 relatively short paragraphs (A, B, C), we think further separate headings are not needed.

**Comment 2.14:** 257-264: These lines are largely a reiteration of the results, which seems unnecessary and better off being cut.
**Answer 2.14:** Thank you for your suggestion. We have rewritten this section of the discussion as following: “*Girls present less often to the ED with fever and respiratory symptoms, and when they do so they have less frequently tachypnea (15% vs 16%) and increased work of breathing (8% vs 12%) compared with boys.* *Few differences in management between boys and girls exist, but we observed consistently lower proportions of girls receiving inhalation medication after adjustment for patient characteristics and markers of disease severity (aOR 0.82, 95% CI 0.74-0.90).”*

**Comment 2.15:** 272: The authors hypothesize that the higher antibiotic prescription rate for girls may be due to a higher rate of urinary tract infections. This hypothesis could have been tested with their data by including a third subgroup for stratification for children with a non-respiratory focus of infection.
**Answer 2.15:** Thank you for your suggestion. We agree that we could have tested our hypothesis and performed the following additional analyses. The higher antibiotic rate in children could be explained by the higher prevalence of UTI in girls, and we have added UTI as a separate focus of infection group in table 1. We adjusted the following sentence in the methods section: *“The focus of infection was allocated retrospectively by the research team. We used the following categories upper respiratory tract, lower respiratory tract, urinary tract and other (gastrointestinal tract, childhood exanthemas/flu-like illness, soft tissue or skin/musculoskeletal, sepsis/meningitis, undifferentiated fever and inflammatory illness).”* The prevalence of UTI in the total group was 1.4% (281/19,781). UTI was more frequently the focus of infection in girls than in boys 2.4% (217/8911) vs 0.6% (64/10,870), with antibiotic treatment in 91% (198/217) in girls and in 89% (57/64) in boys. We have performed a sensitivity analyses where we excluded children with UTI as focus of infection, which showed no significant difference in antibiotic treatment in girls and boys with an aOR 1.03 (0.97-1.10). However, it is not realistic to exclude children with UTI beforehand since in clinical practice you also do not know at initial presentation whether children have an UTI. We added the following to the data analysis section: *“Based on our results, we performed an additional sensitivity analysis with exclusion of children with the urinary tract as focus of infection.”* In the discussion section we added the following: *“This might be explained by a higher prevalence of a urinary tract infection in girls than boys (2.4% vs 0.6%). A sensitivity analysis where we excluded children with a urinary tract infection (N=281) did not show a significant difference in antibiotic prescription in girls and boys (aOR 1.03, 95% CI 0.97-1.10).”* In the results section of our abstract we added the following: *“Girls received less inhalation medication than boys (aOR 0.82, 95% CI 0.74-0.90), but received antibiotic treatment more frequently than boys (aOR 1.09, 95% CI 1.02-1.15), which is associated with a higher prevalence of urinary tract infections.”*
